# The effect of antiemetics in childhood gastroenteritis

**DOI:** 10.1186/1471-2458-13-S3-S9

**Published:** 2013-09-17

**Authors:** Jai K Das, Rohail Kumar, Rehana A Salam, Stephen Freedman, Zulfiqar A Bhutta

**Affiliations:** 1 Division of Women & Child Health, The Aga Khan University, Karachi, Pakistan; 2 Division of Paediatric Emergency Medicine, The Hospital for Sick Children, Toronto, ON, Canada; 3 Global Child Health and Policy, Centre for Global Child Health, The Hospital for Sick Children, Toronto, ON, Canada

## Abstract

**Introduction:**

Diarrheal diseases are the second leading cause of childhood morbidity and mortality in developing countries and an important cause of malnutrition. An estimated 0.75 million children below 5 years of age die from diarrhea. Vomiting associated with acute gastroenteritis (AGE) is a distressing symptom and limits the success of oral rehydration in AGE leading to an increased use of intravenous rehydration, prolonged emergency department stay and hospitalization. In this review we estimate the effect of antiemetics in gastroenteritis in children.

**Methods:**

We conducted a systematic review of all the efficacy and effectiveness studies. We used a standardized abstraction and grading format and performed meta-analyses for all outcomes with more than two studies. The estimated effect of antiemetics was determined by applying the standard Child Health Epidemiology Reference Group (CHERG) rules.

**Results:**

We included seven studies in the review. Antiemetics significantly reduced the incidence of vomiting and hospitalization by 54%. Antiemetics also significantly reduced the intravenous fluid requirements by 60%, while it had a non-significant effect on the ORT tolerance and revisit rates.

**Conclusion:**

Antiemetics are effective for the management of gastroenteritis in children and have the potential to decrease morbidity and mortality burden due to diarrhea, when introduced and scaled up.

## Introduction

Approximately 6.9 million deaths of children under five years occurred in 2011 due to preventable and treatable causes [1]. Diarrheal diseases are a leading cause of childhood morbidity and mortality in developing countries and an important cause of malnutrition. An estimated 0.751 million children below 5 years of age die from diarrhea and 8 out of 10 of these deaths occur in the first two years of life [2]. The incidence of diarrhea has declined from 3.4 episodes/child year in 1990 to 2.9 episodes/child year in 2010 [3] showing that improvements have been observed, but over a greater span of time.

In 1996, The American Academy of Pediatrics (AAP) issued a consensus statement that antiemetic drugs were not recommended in children with gastroenteritis and healthcare providers should be aware of their potential side effects [4]. However in 2003, the Centers for Disease Control (CDC) and Prevention issued a report which stated that ondansetron could be effective in decreasing vomiting and limiting hospital admission [5]. This was endorsed by the AAP in 2004 and although the recommendations do not support the routine use of pharmacologic therapy, the policy states that, ondansetron may be beneficial in limiting vomiting and hospital admissions [6]. Similarly, a European guideline stated that antiemetics might be of value in children with severe vomiting [7].

Vomiting associated with acute gastroenteritis (AGE) is a distressing symptom, both for children and their parents. Furthermore, vomiting limits the success of oral rehydration in AGE leading to an increased use of intravenous (IV) rehydration, need for prolonged emergency department stays and hospitalizations. Thus despite being a subject of controversy, a number of antiemetic agents are now commonly administered worldwide in an attempt to reduce vomiting in children with AGE. These include dopamine (D2) antagonists, serotonin or 5-hydroxytryptamine (5-HT3) antagonists, anticholinergic agents, antihistamines, benzodiazepines, and corticosteroids which are administered orally, intravenously or rectally. Choosing between these therapeutic agents involves careful consideration of a number of factors, including effectiveness, side effect profiles and cost. A national survey conducted in United States of America (USA) estimates that 61% of physicians would administer antiemetics during oral rehydration if they felt it to be necessary [8]. Another survey carried out in Italy, reports that 79% of pediatricians use antiemetics to control vomiting in AGE [9]. Antiemetics such as promethazine, prochlorperazine, and metoclopramide are known to have serious side effects; hence they are less commonly prescribed [10]. Recently antiemetics such as ondansetron have been used in secondary care setting in pediatric population. A number of randomized control trials have been carried out to evaluate its efficacy, safety and cost effectiveness [11-18]. Some researchers have also used rectal dimenhydrinate [19] and dexamethasone [20] but the numbers of studies are limited and clear evidence of any effect on outcomes is yet to be clear.

We conducted a systematic review followed by a meta-analysis to determine whether antiemetic drug use in gastroenteritis provides symptomatic relief and improves other clinically significant outcomes and whether important adverse effects result from using these medications. We have reviewed the available literature and evaluated the quality of included studies according to the Child Health Epidemiology Reference Group (CHERG) adaptation of Grading of Recommendations, Assessments, Development and Education (GRADE) criteria [21]. The review has been designed according to Lives Saved Tools (LiST) and is therefore different from the previously done reviews.

## Methods

**Figure 1:**
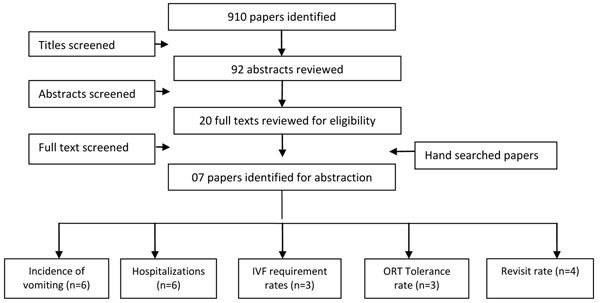
Search strategy flow diagram

We systematically reviewed all published literature until January 2012. A search was conducted in Pubmed, Medline, Cochrane Libraries, EMBASE and World Health Organization (WHO) regional databases to identify all published and unpublished clinical trials, additional studies were identified through hand search of references from included studies (figure 1). We used the Medical Subject Heading Terms (MeSH) and keyword-search strategy using various combinations of: gastroenteritis, vomiting, antiemetics and children. No language or date restrictions were employed in the electronic search. Two authors independently assessed the eligibility using pre-defined inclusion and exclusion criteria and performed data extraction. Any discrepancies between the reviewers in either the decision of inclusion or exclusion of studies or in data extraction were resolved by discussion aimed at reaching consensus.

### Inclusion criteria

We limited the inclusion to randomized and quasi-randomized trials where any antiemetic was administered to children with vomiting associated with AGE. We considered any antiemetic administered orally, intravenously or as a suppository at any dosage, prescribed to terminate or reduce vomiting versus a placebo or nothing. Our initial objective was to evaluate the effectiveness of antiemetics in children aged 0 to 5 years presenting with AGE. However, the literature search did not identify any studies that provided us with data specific to this age group; hence we expanded our eligibility to include studies which had recruited children aged 0 to 12 years. We excluded studies in which patients had vomiting due to alternative etiologies, which were done on adults and which did not have a placebo or a suitable control group.

### Abstraction, analysis and summary measure

All the studies that met the final inclusion criteria were double data abstracted into a standardized form for each outcome of interest. We extracted the following details:

1. Study methods: method of allocation, masking of participants and outcomes, exclusion of participants after randomization and proportion of losses to follow-up.

2. Participants: country, sample size, age, inclusion and exclusion criteria.

3. Intervention: type of antiemetic; dose, frequency and route.

4. Outcomes: any primary and secondary outcomes.

Each study was assessed and graded according to the CHERG adaptation of the GRADE technique [21]. Individual studies were graded according to strengths and limitations of the study. Studies received an initial score of high if a randomized or cluster randomized trial and then the grade was decreased for each study design limitation, if applicable. A study was downgraded if there were limitations in the conduct of studies e.g. inadequate methods of sequence generation or allocation concealment and/or high loss to follow-up (>20%). Risk of bias in the included studies was assessed according to the latest Cochrane Handbook. A grade of “high”, “moderate”, “low” and “very low” was used for grading the overall evidence indicating the strength of an effect on specific health outcome [21].

### Quantitative data synthesis

Outcomes were double data extracted and were analyzed using RevMan version 5.1. The binary measure for individual studies and pooled statistics was reported as the relative risk (RR) between the experimental and control groups with 95% confidence intervals (CI). Mantel–Haenszel pooled RR and corresponding 95% CI were reported or the DerSimonian–Laird pooled RR and corresponding 95% CI where there was an unexplained heterogeneity.

The weights given to each study were based on the inverse of the variance. Heterogeneity was quantified by Chi^2^ and I^2^, which can be interpreted as the percentage of the total variation between studies that is attributable to heterogeneity rather than to chance, a low p-value (less than 0.1) or a large chi-squared statistic relative to its degree of freedom and I^2^ values greater than 50% were taken as substantial and high heterogeneity. In situations of high heterogeneity, causes were explored by sensitivity analysis and random effect models were used.

## Results

We identified 910 papers from the database search. After the initial title and abstract screening, 20 full texts were reviewed to identify papers which met the inclusion criteria and had outcomes of our interest. As no paper reported data exclusively for the 0-5 years age group, we expanded our study population to include children up to 12 years of age. Seven papers [12,13,15,17-20] met our inclusion criteria and had the outcome measures of our interest, were finally selected for abstraction and analysis (table 1). All of these were double blind randomized controlled trials that were conducted in developed nations. Six of the seven studies were conducted in an emergency department (ED) setup while one [19] was in an outpatient setup and was a multicenter study. Children analyzed by these studies varied in age from 5 months to 12 years. Various drugs were used as antiemetics; four trials used oral ondansetron, one used rectal dimenhydrinate, participants of one trial were either given IV ondansetron or IV metoclopramide, and compared against placebo, while participants of one trial were either given IV ondansetron or IV dexamethasone. None of the studies had isolated the cause of AGE or stratified results according to causative agents, although cases with dysentery were excluded from the trials. To estimate the effectiveness of antiemetics and its possible role in gastroenteritis, we found six papers that reported data on vomiting and hospitalization outcomes. Three papers reported on outcomes of Intravenous fluid (IVF) requirements, oral rehydration therapy (ORT) tolerance, IVF requirement rates and admission within 72 hours of discharge from the ED. Table 2 shows the results and quality assessment of studies by outcome.

**Table 1:** Characteristics of included studies

Author	Year of publication	Country	Period of Intervention	Target population	Antiemetic	Route of administration	Dose and Frequency	Duration of Follow up	Study design
Uhlig [19]	2009	Germany	December 2005 to May 2007	Children 6 months to 6 years with suspected infectious gastroenteritis (<24 hours) with mild or no dehydration, 2 vomiting in 12 hours, > 7kg	Dimenhydrinate	Rectal Suppositories	<15kg 40mg, 15 to 25 kg 80mg, >25kg 120 mg	18-24 hours after randomization, and 7-14 days after randomization	Double Blind, Prospective, Randomized, Placebo control, Multicenter
Freedman [13]	2006	USA	January 2004 to April 2005	Children 6 months to 10 years with vomiting or dehydration as a result of AGE and at least one episode of nonbilious vomiting, and no severe dehydration	Ondansetron	Oral	8-15kg 2mg, 15-30kg 4mg, >30kg 8mg	day 3 and day 7 via telephone, last follow-up till max of 2 weeks	Double Blind, Prospective Placebo
Ramsook [15]	2002	USA		Children 6 months to 12 years with clinically diagnosed AGE with 5 episodes of vomits in 24 hours	Ondansetron	Oral	6 months to 1 year 1.6mg, 1 year to 3 years 3.2mg, 4 to 12 years 4mg	24 and 48 hours	Double Blind, Prospective
Roslund [17]	2008	USA	July 1, 2004, to August 1, 2005	Children 1 to 10 years with AGE and mild to moderate dehydration who failed controlled oral challenge in ED	Ondansetron	Oral	<15kg 2mg, 15 to 23 kg 4mg, >30kg 6 mg	1 week	Double Blind, Prospective
Stork [20]	2006	USA	November 1999 and February 2005	Children aged 6 months to 12 years, with more than three episodes of vomiting in the past 24 hours, mild/moderate dehydration, and failed oral hydration. Children with a history or physical examination findings inconsistent with the diagnosis of isolated acute viral gastritis were excluded	Ondansetron or dexamethasone with IVF	IV	dexamethasone 1 mg/kg (15 mg maximum), ondansetron 0.15 mg/kg	2 hour follow-ups up to 48 hours	Double Blind, Prospective
Yilmaz [18]	2010	Turkey	August 2003 and September 2004	Children aged 5 months to 8 years who had nonbillious, nonbloody vomit at least 4 times in the last 6 hours, who could not tolerate ORT, at least four episodes of diarrhea in the previous 24 hours, and who had mild-to-moderate dehydration. Aetiology of acute gastroenteritis (viral, bacterial or amebic) was not taken into account in the patients included in the study.	Ondansetron	Oral	ondansetron 0.2 mg/ kg 8 hourly	24 hours	Double blind, Prospective
Cubeddu [12]	1997	Venezuela		6 months to 8 years with spontaneous vomiting with in 1 hour and no severe dehydration	Ondansetron, metoclopramide	IV	ondansetron 0.3 mg/kg single dose, metoclopramide 0.3 mg/kg single dose	24 hours	Double Blind, Prospective, parallel group, placebo

### Vomiting

**Figure 2:**
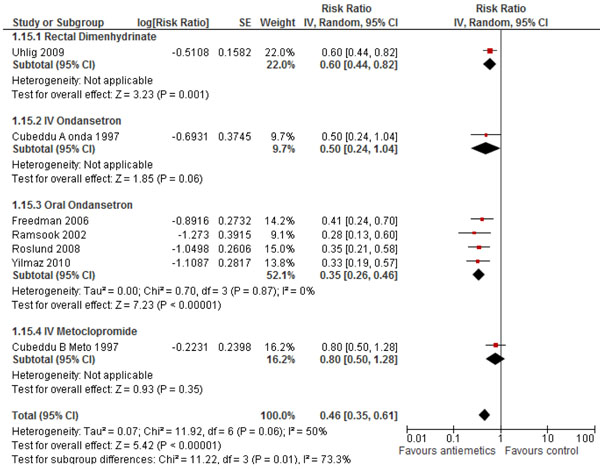
Forest Plot for the effect of antiemetics for the treatment of gastroenteritis on incidence of vomiting

Data on vomiting available from six studies [12,13,15,17-19] and 830 participants was pooled and analyzed for this outcome. Results (Figure 2) indicate that antiemetics were associated with a significant 54% reduction in the incidence of vomiting (RR: 0.46 95% CI: 0.35, 0.61). The follow up periods used for this particular outcome varied across studies. As heterogeneity was high (Chi^2^ = 11.92, I^2^ = 50%, P = 0.06) a random effect model was used. Sub group analysis based on the different antiemetics used, showed that oral ondansetron and rectal dimenhydrinate were associated with a significant reduction of 65% (RR: 0.35, 95% CI: 0.26, 0.46) and 40% (RR: 0.60, 95% CI: 0.44, 0.82) respectively while IV ondansetron and metoclopramide had a non-significant reduction of 50% (RR: 0.50, 95% CI: 0.24, 1.04) and 20% (RR: 0.80, 95% CI: 0.50, 1.28) respectively although only one study was analyzed for all the antiemetics except for oral ondansetron.

### Hospitalization

**Figure 3:**
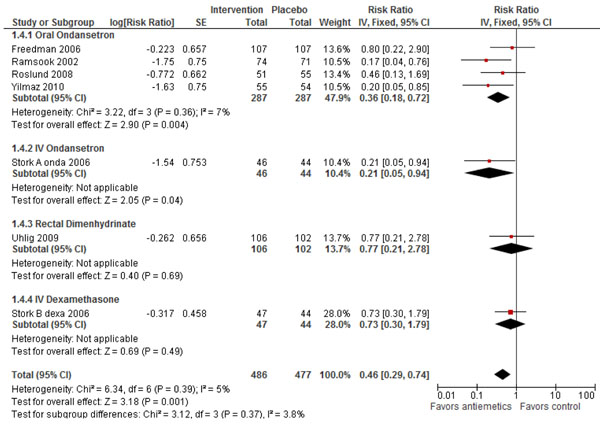
Forest Plot for the effect of antiemetics for the treatment of gastroenteritis on hospitalizations during ED stay

Data from six studies [13,15,17-20] and 963 participants indicated that there was a significant 54% (RR: 0.46, 95% CI: 0.29, 0.74) reduction in the incidence of hospitalization after the use of antiemetics (figure 3). As heterogeneity was low (Chi^2^ = 6.34, I^2^ = 5%, P=0.39) a fixed effect model was used. Subgroup analysis for different antiemetics showed that oral and IV ondansetron significantly reduced the incidence of hospitalization by 64% (RR: 0.36, 95% CI: 0.18, 0.72) and 79% (RR: 0.21, 95% CI: 0.05, 0.94) respectively. While there was a non-significant reduction in the incidence of hospitalization of 23% (RR: 0.77, 95% CI: 0.21, 2.78) and 27% (RR: 0.73, 95% CI: 0.30, 1.79) when rectal dimenhydrinate and IV dexamethasone were used as antiemetics respectively. Hospitalization within 72 hours from discharge from the ED was also reported by three studies and showed that oral ondansetron had a non-significant 34% (RR: 0.66, 95% CI: 0.37, 1.19) reduction.

### Revisit rate

Four studies [13,15,17,18] evaluated the revisit rates with use of oral ondansetron with a total of 553 participants and indicated that oral ondansetron reduced the revisit rates to the ED by a non-significant 3% (RR: 0.97, 95% CI:0.62, 1.53)**.** There was no significant heterogeneity and hence a fixed effect model was used for analysis.

### IVF requirement rate

IVF requirement rate were analyzed in two ways by the studies included; first, if the patient required IVF during the stay in ED and secondly if the patient required IVF within 72 hours of discharge from the ED. Three studies [13,15,17] reported both the outcomes and evaluated the effect of oral ondansetron on IVF requirement rates. Based on the analysis from the datasets of these three studies, oral ondansetron reduced the IVF requirements during the ED stay by 60% (RR: 0.40, 95% CI: 0.29, 0.56) and within 72 hours of discharge from ED by 34% (RR: 0.66, 95% CI: 0.37, 1.19). A fixed effect model was used for analysis as there was low heterogeneity.

### ORT tolerance rate

Tolerance to ORT as an outcome was reported by three studies [17,18,20] and it indicates a significant 22% (RR: 1.22, 95%CI: 1.01, 1.46) increase in tolerance after the use of antiemetics**.** Subgroup analysis for different antiemetics shows that oral ondansetron is associated with a 33% increase (RR: 1.33 95% CI: 0.98, 1.80), IV ondansetron with a 29% increase (RR: 1.29 95% CI: 1.01, 1.63) and IV dexamethasone was associated with a non-significant 8% reduction (RR: 0.92, 95% CI: 0.67, 1.26) in ORT tolerance rates.

### Recommendation for the LiST model

**Figure 4:**
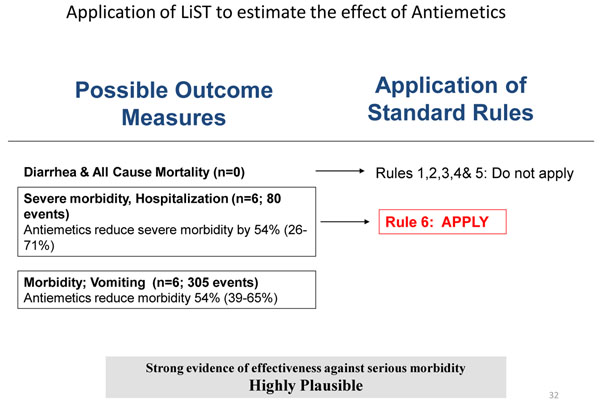
Application of standardized rules for choice of final outcome to estimate effect of antiemetics in gastroenteritis

We applied the CHERG rules for evidence review to the outcomes assessed for the effect of antiemetics on gastroenteritis in children. As there was no data on mortality; either all-cause or cause specific, we used a severe morbidity outcome to estimate the effect on mortality. The six RCTs reported a 54% decrease in hospitalization rates for about 80 admissions. The results also report a 54% reduction in episodes of vomiting after the use of antiemetics for 300 episodes of vomiting. As the studies included did not isolate the specific cause of AGE and we cannot stratify our data according to type of diarrhea thus these results are applicable to all cases of AGE, excluding dysentery. Hence we estimate and propose a 54% reduction in diarrhea related mortality with the use of antiemetics in cases of diarrhea associated with vomiting. (see Figure 4)

## Discussion

Vomiting continues to be associated with hospitalization, use of IVF, and significant morbidity among AGE affected children worldwide. Recently the role of antiemetics for controlling vomiting in children has been debated. Clinical practice guidelines for the treatment of children with gastroenteritis recommend supportive care using ORT for mild to moderate dehydration, without fully endorsing the use of antiemetic medications to control vomiting. However in clinical practice, it appears that antiemetics are widely popular among physicians [8,22].

Our analysis of the effect of antiemetics in gastroenteritis suggests that antiemetics are likely to be beneficial in children with vomiting. The RCTs included in our study looked at ondansetron, metoclopramide, dimenhydrinate and dexamethasone. The administration of oral ondansetron was found to reduce incidence of vomiting, reduction in hospitalization and IVF requirements while IV ondansetron was associated with a significant increase in tolerance to ORT. Rectal dimenhydrinate also significantly reduced the incidence of vomiting but has only been evaluated by a single study. There is insufficient evidence to support the role of IV metoclopramide in children with AGE as far as the incidence of vomiting is concerned but significantly reduced the hospitalization rates. Rectal dimenhydrinate also significantly reduced the incidence of vomiting and IV dexamethasone did not significantly decrease hospitalization rates or increased tolerance to ORT. The outcomes and recommendation from the meta-analysis are summarized in table 2 based on the LiST model suggested by the CHERG reviews of intervention effectiveness on child survival [23].

**Table 2:** Quality assessment of trials of antiemetics on vomiting and hospitalization rates in acute gastroenteritis

Quality Assessment						Summary of Findings		
				**Directness**		**No of events**		

**No of studies**	**Design**	**Limitations**	**Consistency**	**Generalizability to population of interest**	**Generalizability to intervention of interest**	**Intervention**	**Control**	**Relative Risk (95% CI)**

***Morbidity-Vomiting: Moderate outcome-specific quality***								

6 [12,13,15,17-19]	RCT	Studies used different follow up periods. Random effect model was used	All studies suggest benefit	Four of six studies were conducted in developed countries	Pooled results for different types of antiemetics and route of administration.	95	210	**0.46 [0.35, 0.61]**^**b**^
4 [13,15,17,18]	RCT		All studies suggest benefit	Three of four studies were conducted in developed countries	Effect of oral ondansetron	47	134	**0.35 (0.26, 0.46)^a^**
1 [12]	RCT	Insignificant effect		In developing country	Effect of IV ondansetron	5	10	**0.50 (0.24, 1.04)**
1 [12]	RCT	Insignificant effect		In developing country	Effect of IV metoclopramide	8	10	**0.80 (0.50, 1.28)**
1 [19]	RCT			In developed country	Effect of rectal dimenhydrinate	35	56	**0.60 (0.44, 0.82)**

***Morbidity- Hospitalization rates: Moderate outcome-specific quality***								

6 [13,15,17-20]	RCT		All studies suggest benefit. Fixed effect model used	Five of six studies were conducted in developed countries	Pooled results for different types of antiemetics and route of administration.	24	56	**0.46 [0.29, 0.74]^a^**
4 [13,15,17,18]	RCT		All studies suggest benefit	Three of four studies were conducted in developed countries	Effect of oral ondansetron	11	33	**0.36 (0.18, 0.72)^a^**
1 [20]	RCT			Developed country	Effect of IV ondansetron	2	9	**0.21 (0.05, 0.94)**
1 [20]	RCT	Insignificant effect		Developed country	Effect of IV dexamethasone	7	9	**0.73 (0.30, 1.79)**
1 [19]	RCT	Insignificant effect		Developed country	Effect of rectal dimenhydrinate	4	5	**0.77 (0.21, 2.78)**

***Revisit rates : low outcome-specific quality***								

4 [13,15,17,18]	RCT	Variable time periods used in the four studies	Two studies suggest benefit while two studies report otherwise	Three out of four studies were conducted in developed countries	All studies used oral ondasetron	34/284	30/269	**0.97 [0.62, 1.53]^a^**

***IVF required rates: Low outcome-specific quality***								

3 [13,15,17]	RCT		All studies are consistent in the results. Fixed effect model used	All studies were conducted in developed countries	All studies used oral ondansetron	35	93	**0.40 [0.29, 0.56]^a^**

***ORT tolerance rates: Low outcome specific quality***								

3 [17,18,20]	RCT		Random effect model used. Two of the three studies suggest benefit	All studies were conducted in developed countries	Pooled results for different antiemetics and routes of administrations	155	125	**1.22 [1.01, 1.46]^b^**

***Admission Rates within 72 hours of discharge from ED: Low outcome-specific quality***								

3 [13,15,17]	RCT		All studies suggest benefit	All studies were conducted in developed countries	All studies used oral ondansetron	18	30	**0.66 [0.37, 1.19]**

***IVF requirement Rates within 72 hours of discharge from ED oral ondansetron: low outcome-specific quality***								

3 [13,15,17]	RCT		Consistent benefit suggested by the three studies	All studies were conducted in developed countries	All studies used oral ondansetron	49	86	**0.57 [0.42, 0.76]**^**a**^

a: Fixed Effect Model

b: Random Effect Model

Oral ondansetron given as a single dose of 0.1-0.25 mg/kg [13,17] in the emergency department to children with mild to moderate dehydration decreases the number of children with persistent vomiting. It also decreases the number of children requiring IV rehydration and hospital admissions. Higher doses have also been used but any added advantage is not apparent [15]. Oral ondansetron may be useful as an adjunctive measure to ORT in the outpatient or primary care setting as well, although no study is available at the moment to confirm this. Thus, it should be an important area of future research.

Adverse effects reported with use of ondansetron were limited to increased mean episodes of diarrhea noticed in a few studies, although this was statistically non-significant. A possible explanation could be the variable duration of follow-up periods employed by different researchers, while it would be ideal for researchers to report the actual output volume of stool rather than the number of episodes [24], which is usually impractical in outpatient clinical trials. The use of rectal dimenhydrinate was not associated with increased episodes of diarrhea or any other major adverse effects [19]. Other antiemetic medications such as metoclopramide appears to be less efficacious in the treatment of gastroenteritis induced vomiting and are associated with more adverse events than ondansetron.

The cost effectiveness of ondansetron has been another area of concern. However, an economic analysis [14] in the USA, the administration of ondansetron to eligible children would prevent approximately 29,246 IV insertions and 7,220 hospitalizations annually. It also reported that at the current average wholesale price, its routine administration to eligible children would annually save society US$65.6 million (US$49.1–US$81.1) and health care payers US$ 61.1 million (US$46.2–US$76.3).

Currently, online pharmacies charge $20 to $30 per pill for the brand name Zofran [25]. Although this seems expensive, if taken into the account the fact that use of single dose of ondansetron reduces IVF requirements and admission rates, it may be extremely cost effective. This might not be the case in developing countries where a majority of children are treated at facilities that utilize minimal resources and cannot afford expensive medications. Hence a very clear evidence of its efficacy and safety should be available before it could be introduced as a standard of care in the clinical guidelines, especially for both developing and underdeveloped countries.

The findings in this systematic review are to a large extent in agreement with those reported by the Cochrane reviews [26] and previously done meta-analysis on the same subject [27-31]. Although we differ from the previously carried out reviews as we have only included studies looking at children aged 0 to 12 years and we have evaluated the quality of the studies and the outcomes based on the LiST model as suggested by CHERG intervention review process. We have also based our recommendations on the CHERG intervention review process [21].

A number of limitations can be observed from the included studies that extend to this review. Firstly the use of antiemetics was studied in the ED setting only while the role of oral antiemetics such as oral ondansetron in outpatient or primary care settings is yet to be evaluated. Other study limitations include that a wide age range of participants were included by different studies and there was no widely agreed-on definitions of the outcome measures (e.g. incidence of vomiting, hospital admission, need for IV rehydration) used by different studies and children were included with various degrees of dehydration.

Currently we need more evidence on safety and efficacy of antiemetics in AGE. A study currently underway will be assessing the use of ondansetron compared with domperidone in children [32]. Studies also need to be done in primary care settings with evaluations of cost effectiveness especially in developing countries. In the meantime, recommendations by the CDC focus on appropriate fluid, electrolyte and nutritional therapy in all patients [5] and if vomiting continues with the possibility of IV rehydration, clinicians should consider using oral ondansetron.

## Conclusion

Antiemetics are effective for the management of gastroenteritis in children and have the potential to decrease the morbidity and mortality burden due to diarrhea, when introduced and scaled up.

## List of abbreviations

AAP: American Academy of Pediatrics; AGE: Acute gastroenteritis; CDC: Centers for Disease Control; CHERG: Child Health Epidemiology Reference Group; CI: confidence intervals; ED: Emergency Department; GRADE: Grading of Recommendations, Assessments, Development and Education; IV: Intravenous; IVF: Intravenous fluid; LiST: Lives Saved Tools; MeSH: Medical Subject Heading Terms; ORT: Oral Rehydration Therapy (ORT); RCTs: Randomized Controlled Trials; RR: Relative Risk; USA: United States of America; WHO: World Health Organization.

## Competing interests

The authors declare no conflict of interests.

## Authors' contributions

Dr ZAB was responsible for designing the review and co-ordinating the review. JKD, RK and RAS were responsible for: data collection, screening the search results, screening retrieved papers against inclusion criteria, appraising quality of papers, abstracting data from papers, entering data into RevMan, analysis and interpretation of data and writing the review. ZAB and JKD critically reviewed and modified the manuscript in addition to Stephen Freedman.

## Declarations

The publication costs for this supplement were funded by a grant from the Bill & Melinda Gates Foundation to the US Fund for UNICEF (grant 43386 to "Promote evidence-based decision making in designing maternal, neonatal, and child health interventions in low- and middle-income countries”). The Supplement Editor is the principal investigator and lead in the development of the Lives Saved Tool (LiST), supported by grant 43386. He declares that he has no competing interests.

This article has been published as part of *BMC Public Health* Volume 13 Supplement 3, 2013: The Lives Saved Tool in 2013: new capabilities and applications. The full contents of the supplement are available online at http://www.biomedcentral.com/bmcpublichealth/supplements/13/S3.
